# Heavy Menstrual Bleeding in Women Treated with Direct Oral Anticoagulants: Results of the Prospective HEMBLED Registry

**DOI:** 10.1055/a-2724-4458

**Published:** 2025-11-12

**Authors:** Edelgard Lindhoff-Last, Inka Wiegratz, Olivia Ott, Yvonne Weil, Christoph Sucker, Susan Halimeh, Holger Seidel, Christian Schambeck, Konstantin Kirchmayr, Eva Herrmann

**Affiliations:** 1 Cardioangiology Center Bethanienhospital (CCB), Department of Coagulation Disorders, Frankfurt, Germany; 2 Fertility Center Am Palmgarten, Department of Obstetrics and Gynecology, Frankfurt, Germany; 3 Praxisklinik in Mittelhessen, Department of Angiology, Wetzlar, Hessen, Germany; 4 Coagumed Gerinnungszentrum, Department of Haemostaseology, Berlin, Germany; 5 Coagulation Center Rhein-Ruhr, Department of Haemostaseology, Duisburg, Germany; 6 Center for Blood Coagulation Disorders and Transfusion (CBT), Department of Haemostaseology, Bonn and Dortmund, Germany; 7 Haemostasikum, Department of Haemostaseology, München, Germany; 8 Punktmed Medical Care Center, Department of Angiology and Haemostaseology, Amberg and Nürnberg, Germany; 9 Institute of Biostatistics and Mathematical Modelling, Goethe University Frankfurt, Frankfurt, Germany

**Keywords:** heavy menstrual bleeding, venous thrombosis, direct oral anticoagulants, rivaroxaban, apixaban

## Abstract

**Background:**

Heavy menstrual bleeding (HMB) is a common complication of anticoagulant therapy in menstruating women with venous thromboembolism (VTE). Direct oral anticoagulants (DOAC) used for VTE treatment may differ in their menstrual bleeding profiles. Therefore, the prospective multicenter noninterventional investigator-initiated HEMBLED registry ( **he** avy **m** enstrual **ble** eding in patients treated with **D** OAC) was performed to analyze spontaneous menstrual bleeding in women treated with therapeutic DOAC doses.

**Methods:**

A modified pictorial blood assessment chart (PBAC) score was used to define the severity of menstrual bleeding. Patients were only included when they did not use hormonal or intrauterine contraception methods. The prospective follow-up was 4 months. The primary endpoint was the comparison of the PBAC scores between the individual DOAC groups.

**Results:**

Overall, 73 patients with 213 monthly assessments of the PBAC scores were analyzed. Patients were on average 35 years old and were anticoagulated with apixaban (62%), rivaroxaban (26%), edoxaban (7%), or dabigatran (6%). The PBAC scores of the rivaroxaban group (mean: 145 points) were significantly increased by 54% compared with the apixaban group (mean: 93 points, *p* = 0.0193). HMB (PBAC score > 100 points) at least once was detected in 53% of the apixaban group compared with 79% of the rivaroxaban group ( *p* = 0.0913). The duration of menstrual bleeding was numerically shorter in the apixaban group compared with the rivaroxaban group ( *p* = 0.1894).

**Conclusion:**

DOAC differ in their influence on the intensity of spontaneous menstrual bleeding. This should be taken into account when advising young women with VTE who need an oral anticoagulant.

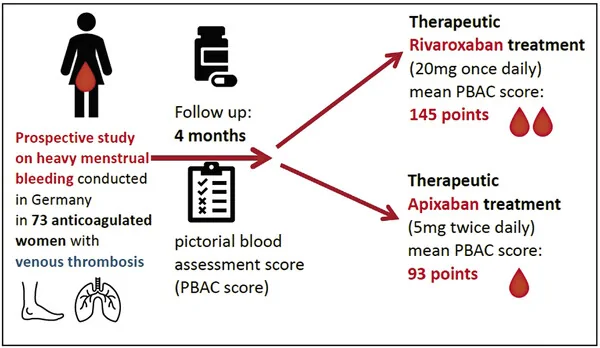

## Introduction

Heavy menstrual bleeding (HMB) is a common complication of anticoagulant therapy in premenopausal women with venous thromboembolism (VTE). Up to 66% of women will develop HMB after the start of therapeutic anticoagulation based on an objective measurement of menstrual blood loss. 1 It impacts quality of life and can lead to premature cessation of anticoagulation. 2 The prevalence of HMB in women treated with oral anticoagulants is significantly higher than in women who are not anticoagulated. 3 During treatment with anticoagulants, the mean duration of menstrual bleeding can increase from 5.6 to 6.1 days. 4

So far, prior to the introduction of the direct oral anticoagulants (DOAC) era, little has been published on this topic. 3 With the rapidly increasing use of DOAC for treatment of VTE during the last decade, there is increasing data to suggest that DOAC differ in their menstrual bleeding profiles. 5 6 7 8 9 10 11 These findings come from retrospective or prospective single-center studies and post hoc analysis of regulatory studies, in which HMB has not been a predefined safety outcome. In addition, in most of these publications, there is a lack of information about the use of different contraceptive methods, which can influence HMB. Another limitation is the various definitions of HMB, which makes comparison between studies regarding the incidence of HMB difficult. 2

Therefore, the prospective multicenter noninterventional investigator-initiated HEMBLED registry ( **he** avy **m** enstrual **ble** eding in patients treated with **D** OAC) was performed in eight German outpatient coagulation centers from 2020 to 2024 to analyze HMB in women (18–50 years) treated with DOAC who do not use hormonal contraceptives or intrauterine devices, because these measures can influence the intensity and duration of menstrual bleeding. 2

## Methods

The HEMBLED registry was a German prospective, open, multicenter cohort study including female outpatients of reproductive age who were anticoagulated with therapeutic doses of either apixaban, rivaroxaban, edoxaban, or dabigatran because of VTE. Patients were included from 2020 to 2024. The primary aim was to evaluate and compare the incidence of HMB in women treated with DOAC using a modified pictorial blood assessment chart (PBAC)-score 2 12 : a version translated into German was used for better understanding. In addition, different numbers of blood drops instead of pictograms were used to better define the different sizes of tampons and pads and thus also the amount of menstrual blood loss ( Supplementary Material , available in the online version only). During their first visit, patients were instructed by the treating physician or a study nurse on how to use the PBAC score. During the follow-up visit, the completed PBAC score sheets were collected and checked together with the individual patient for completeness and correct calculation of the scores per menstrual bleeding.

The study protocol, any amendments, and the subject informed consent received independent local Ethics Committee approval from all German participating centers before initiation of the study. Patients were recruited at eight outpatient coagulation centers in Germany from 2020 to 2024. All patients provided written informed consent.

### Patients

We included consecutive menstruating women aged 18 to 50 years with objectively confirmed symptomatic first or recurrent VTE who fulfilled the following inclusion criteria and none of the exclusion criteria presenting to one of the participating outpatient coagulation centers: inclusion criteria were regular menstrual bleeding, treatment with maintenance therapeutic doses of apixaban (5 mg twice daily), rivaroxaban (20 mg once daily), dabigatran (150 mg twice daily) or edoxaban (60 mg once daily) for at least 7 days before inclusion and planned continued therapeutic anticoagulation with apixaban 5 mg twice daily, rivaroxaban 20 mg once daily,, dabigatran 150 mg twice daily or edoxaban 60 mg once daily for at least the following next 4 months after inclusion.

All included patients who had developed VTE while using oral contraceptives had already stopped hormonal contraception at least 25 days before they were included in the registry.

Key exclusion criteria were patients with antiphospholipid-syndrome, patients with a past history of hysterectomy or ovarectomy, patients reporting HMB without anticoagulation in their past history, patients with intake of hormonal contraceptives, hormone replacement therapy, use of intrauterine devices (IUD, either copper or hormone releasing), contraindications to treatment with DOAC and treatment with rivaroxaban (10 mg or 15 mg once daily) or apixaban (2.5 mg twice daily) in reduced dosages.

### Study Procedures

Study enrollment was performed during therapeutic treatment with a DOAC, which was expected to last at least 4 months after inclusion in the registry.

**Fig. 1:**
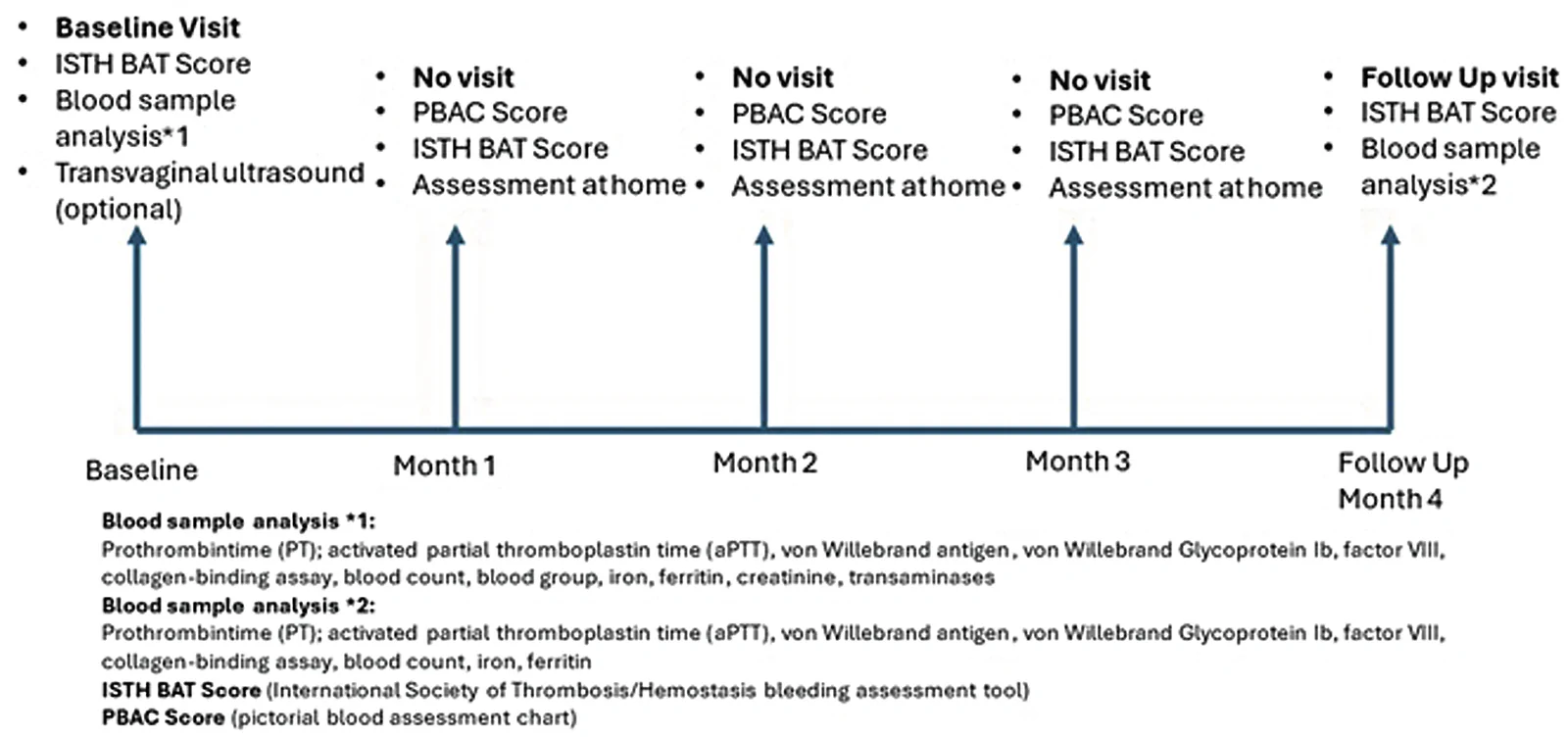
Schedule of assessments.

After inclusion, a baseline visit was performed. The bleeding history of the patients was systematically analyzed by use of a modified International Society of Thrombosis and Hemostasis (ISTH) Bleeding Assessment Tool (BAT)-Score 2 13 with a cut-off for abnormal bleeding ≥ 6 points in women. Four months after inclusion, a follow up visit (visit 1) was planned ( Fig. 1 ). Laboratory samples were taken at the baseline visit and at the follow up visit (prothrombin time, activated partial thromboplastin time, von Willebrand antigen, von Willebrand Glycoprotein Ib, factor VIII with DOAC stop, a collagen binding assay, a blood count, blood group, iron, ferritin, and creatinine, and transaminases only at baseline; Fig. 1 ). In addition a transvaginal ultrasound was offered free of charge (optional) to detect possible uterine causes (i.e., uterine fibroids, adenomyosis, endometrial polyps) of menorrhagia.

Patients received three PBAC score Sheets to document the intensity of the menstrual bleeding of three consecutive menstrual bleeding cycles at home. In addition, they received three ISTH BAT-Score Sheets to document monthly whether different types of bleeding, in addition to menstrual bleeding, had occurred in the meantime. Patients were advised to visit their local gynecologist in case of HMB during follow up and results of these investigations were additionally documented in the case report form (CRF). At the follow-up visit, the results of the completed PBAC score sheets and ISTH BAT-score sheets were collected and added to the CRF.

### Outcomes

The primary outcome was the incidence of HMB at least once during the follow-up period. HMB was defined as a PBAC score > 100 points, which is indicative of a blood loss > 80 mL. 12 14

Main secondary outcome was the prevalence of von Willebrand's disease, further secondary objectives were to assess the correlation of HMB with von Willebrand's disease, age, blood group, underlying uterine pathologies, that is, presence of uterine fibroids, endometrial polyps and/or adenomyosis (only in patients with transvaginal ultrasound), iron deficiency, hemoglobin level and documentation of use of nonsteroidal anti-inflammatory drugs (NSAID).

### Statistical Analysis

When planning the registry, sample size estimation was based on a binary primary endpoint. The following primary objective was planned:

Comparison of the frequency of HMB (occurrence at any time point using the PBAC score > 100) between the different DOAC groups using a two-sided exact Fisher test with a significance level of *α* = 5%. If score ratings were missing at some time points (i.e., in patients who only presented with one or two menstrual bleeding cycles during 4 months of follow-up), the primary endpoint was defined on the available assessment.

At the time when the registry was planned in 2018, data on HMB during treatment with DOAC were scarce. At that time, there was only one prospective cohort study available, which compared the occurrence of HMB in patients treated with apixaban or rivaroxaban. 5 The results of this investigation were used for the original sample size calculation, which used the assumption of rates of HMB of 25% in the rivaroxaban group and of 9% in the apixaban group. Data on the occurrence of HMB during treatment with edoxaban and dabigatran were not available at that time. Therefore, rates were calculated to be 9% for dabigatran (intake twice daily, similar to apixaban) and 25% for edoxaban (intake once daily, similar to rivaroxaban). Simulations showed that the power was at least 80% if 237 patients were included (assuming 101 patients in the apixaban and 101 patients in the rivaroxaban groups, respectively, and 35 patients in the edoxaban and dabigatran groups). To account for some missing data or dropouts, it was planned to prospectively include 250 patients.

Due to the COVID-19 pandemic, the start of the registry was delayed, and the first patient was included in October 2020 instead of April 2020. In addition, some of the local ethical committees stopped working for some time, which delayed the initiation of some of the planned study centers. Besides, many study centers were not able to include new patients because of the COVID-19 pandemic, even in the following years. Recruitment of patients slowed down and was therefore expected to remain reduced in comparison to the initial study plans. For these reasons, it was decided in 2022 to extend the recruitment time until December 2023 instead of August 2022 and to reduce the patient number from 250 to 150 patients. The power would have been 61% if the reduced number of 150 patients were included (assuming approximately 65 patients in the apixaban and 65 patients in the rivaroxaban groups and approximately 20 patients in the edoxaban and dabigatran groups). Nevertheless, recruiting time was limited. As there were strict inclusion and exclusion criteria to ensure a homogeneous population, finally only 45 women were included in the apixaban group, 19 women in the rivaroxaban group, and five and four patients in the edoxaban and dabigatran groups, respectively (total: *n* = 73 DOAC-treated patients). This was mainly due to the fact that patients were only included when they did not need or want oral contraceptives or IUDs, despite the fact that they were informed by the treating physicians that safe contraception was mandatory during DOAC treatment. Therefore, only a power of 35% for the original primary aim was reached when comparing the binary primary endpoint. Therefore, before the export of the final data for statistical analysis, it was decided to change the primary objective and to use the PBAC score without dichotomization to obtain a larger power. This updated primary objective was a comparison of the PBAC scores between the different DOAC treatment groups using a linear mixed effect model with a hierarchical approach: the apixaban and rivaroxaban groups were compared first, and as a secondary analysis, a comparison between the apixaban group and either the edoxaban group or the dabigatran group was performed. A log-transformation was used where appropriate, and a significance level of *α* = 5% was applied. In addition, a multivariate linear mixed-effect regression model of the nondichotomized PBAC scores was used to assess the difference in the primary endpoint between the DOAC groups with a special focus on comparing the rivaroxaban and apixaban groups, while accounting for further factors. Because of the small sample size, the multivariate analysis had to focus on the most important factors. In addition, the originally planned dichotomized endpoint analysis (PBAC score > 100 points at least once during follow-up) was evaluated with the exact Fisher test with a significance level of *α* = 5%.

As originally planned, further descriptive and explorative analysis was performed comparing secondary endpoints between the groups with appropriate univariate and multivariate statistical tests with focus on the exact Fisher test and Mann–Whitney *U* test. ISTH scores were analyzed with ordinal mixed-effect regression.

Post hoc, we performed a sensitivity analysis of the impact of six patients with a known change in anticoagulant treatment because of increased menstrual bleeding before inclusion in the registry and a repeated correlation analysis of the association between hemoglobin and hemoglobin decrease and PBAC scores.

For categorial variables, absolute and relative frequencies were analyzed. For continuous variables, the mean and standard deviation or % were given. Pairwise comparisons of reassessments were performed with the Wilcoxon signed-rank test. All tests were two-sided and used a significance level of *α* = 5%.

The statistical analysis was performed with R version 4.4.1 (R Foundation for Statistical Computing) using the basic packages, doBy, nlme, rmcorr, and lme4.

## Results

From October 2020 to April 2024, 73 patients with confirmed symptomatic first or recurrent VTE and active menstrual cycles, with a mean age of 35 years, were included. At baseline, 45 of 73 patients received apixaban (62%), 19 patients received rivaroxaban (26%), five patients received edoxaban (7%), and four patients received dabigatran (6%). Baseline characteristics are summarized in Table 1 .

**Table 1:** Baseline characteristics of 73 female patients of reproductive age with venous thromboembolism (VTE)

Variable	All ( *n* = 73)	Apixaban ( *n* = 45)	Rivaroxaban ( *n* = 19)	Edoxaban ( *n* = 5)	Dabigatran ( *n* = 4)
Age (y)	35 ± 9.1	34 ± 8.7	35 ± 10.6	34 ± 10.2	40 ± 4.9
BMI (kg/m ^2^ )	28.2 ± 7.7	27.4 ± 7.1	30.3 ± 6.5	24.9 ± 6.2	32.2 ± 18.0
ISTH bleeding score	1.0 ± 1.2	0.9 ± 1.3	0.9. ± 0.9	1.4 ± 1.3	1.3 ± 1.9
Index VTE a					
DVT	42 (58%)	27 (60%)	11 (58%)	4 (80%)	0 (0%)
PE	22 (30%)	13 (29%)	6 (32%)	2 (40%)	1 (25%)
CVT	8 (11%)	3 (7%)	1 (5%)	0 (0%)	4 (100%)
AVT	5 (7%)	4 (9%)	1 (5%)	0 (0%)	0 (0%)
ICVT	2 (3%)	2 (4%)	0 (0%)	0 (0%)	0 (0%)
ST	1 (1%)	1 (2%)	0 (0%)	0 (0%)	0 (0%)
SVT	1 (1%)	0 (0%)	1 (5%)	0 (0%)	0 (0%)
MVT	1 (1%)	1 (2%)	0 (0%)	0 (0%)	0 (0%)
Hormonal contraception at the time of thrombosis	32 (44%)	18 (40%)	10 (53%)	3 (60%)	1 (25%)
Regular menstruation	72 (99%)	44 (98%)	19 (100%)	5 (100%)	4 (100%)
Cycle length (d)	26 ± 9.5	25 ± 11.4	27 ± 6.0	27 ± 1.7	28 ± 1.0
Family history of bleeding	4 (5%)	2 (4%)	1 (5%)	1 (20%)	0 (0%)
Use of NSAIDs	10 (14%)	5 (11%)	4 (21%)	0 (0%)	1 (25%)

Abbreviations: AVT, arm vein thrombosis; BMI, body mass index; CVT, cerebral vein thrombosis; DVT, deep vein thrombosis; ICVT, inferior caval vein thrombosis; ISTH, International Society of Thrombosis and Hemostasis; MVT, muscle vein thrombosis; NSAID, nonsteroidal anti-inflammatory drugs; PE, pulmonary embolism; ST, splanchnic vein thrombosis; SVT, superficial vein thrombosis; VTE, venous thromboembolism.

Note: Mean ± standard deviation or % are given.

a Multiple index VTEs in a single patient were possible.

The majority of patients presented with deep vein thrombosis (58%), followed by pulmonary embolism (30%). Overall, 32 patients (44%) had been treated with hormonal contraception at the time of thrombosis but had already stopped the hormonal contraception at least 25 days (median: 196 days, maximum: 18 years) before inclusion in the registry.

In 18% of the patients (13/73), anticoagulation was changed after the thromboembolic event but before inclusion in the registry. Nine of the 13 patients were changed from rivaroxaban to apixaban. Reasons for these changes prior to inclusion were a bleeding event ( *n* = 6), a single positive lupus anticoagulant measurement ( *n* = 1), nausea ( *n* = 1), and preference for an anticoagulant with twice daily dosing ( *n* = 1). All six patients with a bleeding event during rivaroxaban treatment reported on enhanced menstrual bleeding, which had been the reason for the switch to apixaban, but one of these patients changed back to rivaroxaban before study inclusion.

In the four remaining patients, early changes in anticoagulation before inclusion in the registry were observed: one patient switched from apixaban to rivaroxaban because of a suspected but not confirmed recurrent thrombosis. Another patient was changed from apixaban to edoxaban because of nausea and abdominal discomfort. A third patient was switched from edoxaban to apixaban because of an allergic reaction. The fourth patient was first treated with enoxaparin because of breastfeeding and was switched to apixaban after stopping breastfeeding before inclusion in the registry.

After inclusion into the registry, further changes in anticoagulation during the 4-month study period were documented. Immediately after inclusion, one patient switched from edoxaban to dabigatran because of the patient's wish, although there were no documented side effects of edoxaban treatment. One patient was switched by the treating physician from rivaroxaban to apixaban after 1 month because of menorrhagia, and one patient stopped the anticoagulation with rivaroxaban because of menorrhagia after 2 months of follow-up. One further patient stopped edoxaban treatment after the second month of follow-up because of menorrhagia. In addition, two apixaban-treated patients had missing data for 1 or 2 months of follow-up.

Eleven percent of the patients had blood group O (8/71; 95% confidence interval [CI]: 5–21%). None of the women had thrombocytopenia, and one patient was diagnosed with von Willebrand disease type I (1/73, 1.4%).

### Incidence of HMB Depending on DOAC Treatment

**Fig. 2:**
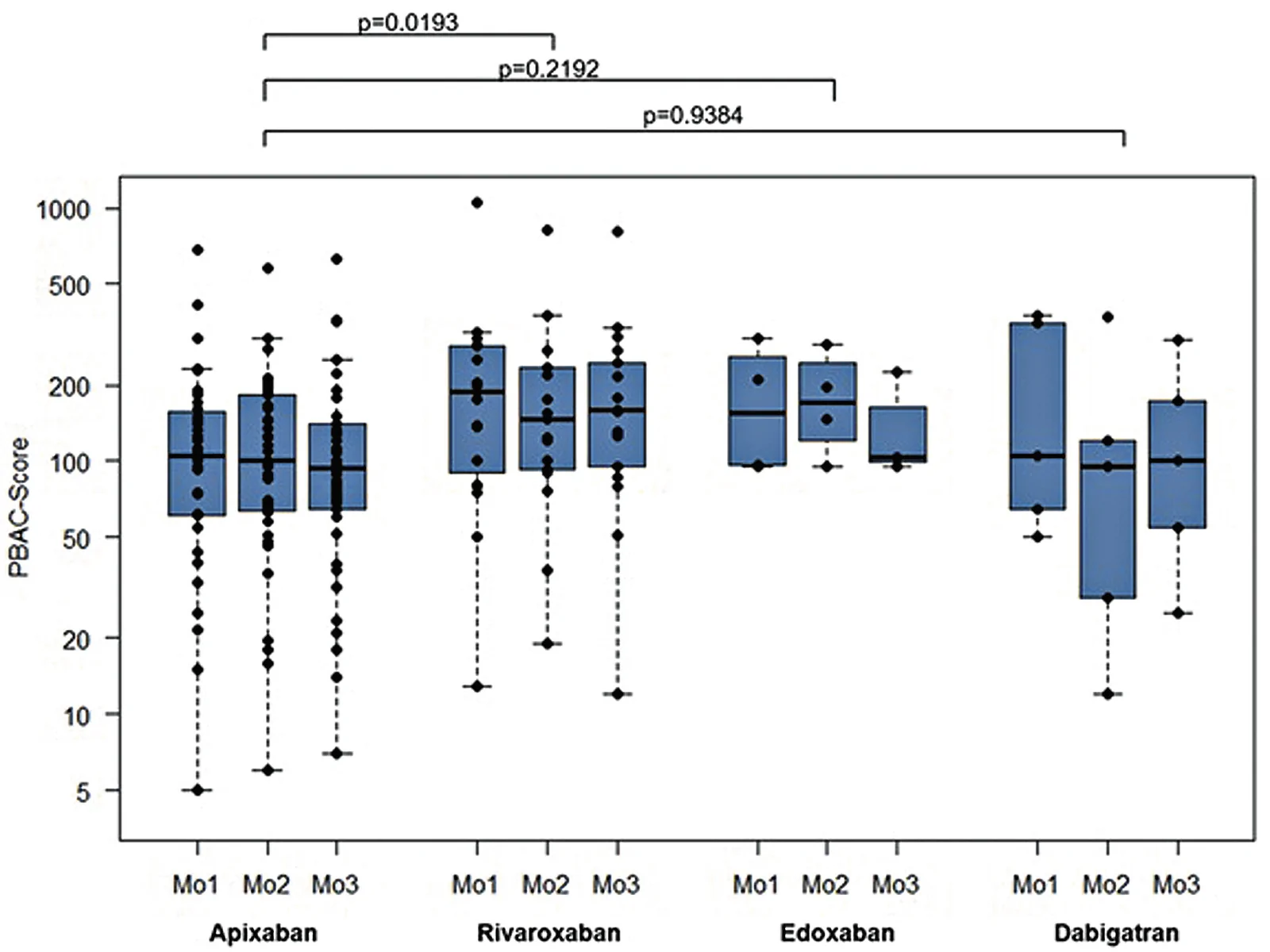
Boxplots of the nondichotomized PBAC score evaluations of the four direct oral anticoagulant (DOAC) treatment groups. Comparisons were performed using a linear mixed effect regression model ( *p* -values) which also provides mean values and 95% CIs: apixaban-group mean: 93 points (95% CI: 73–118), rivaroxaban-group mean: 145 points (95% CI: 103–204), edoxaban-group mean: 161 points (95% CI: 68–381), dabigatran-group mean: 95 points (95% CI: 44–206). Mo, month; PBAC, pictorial blood assessment chart.

**Fig. 3:**
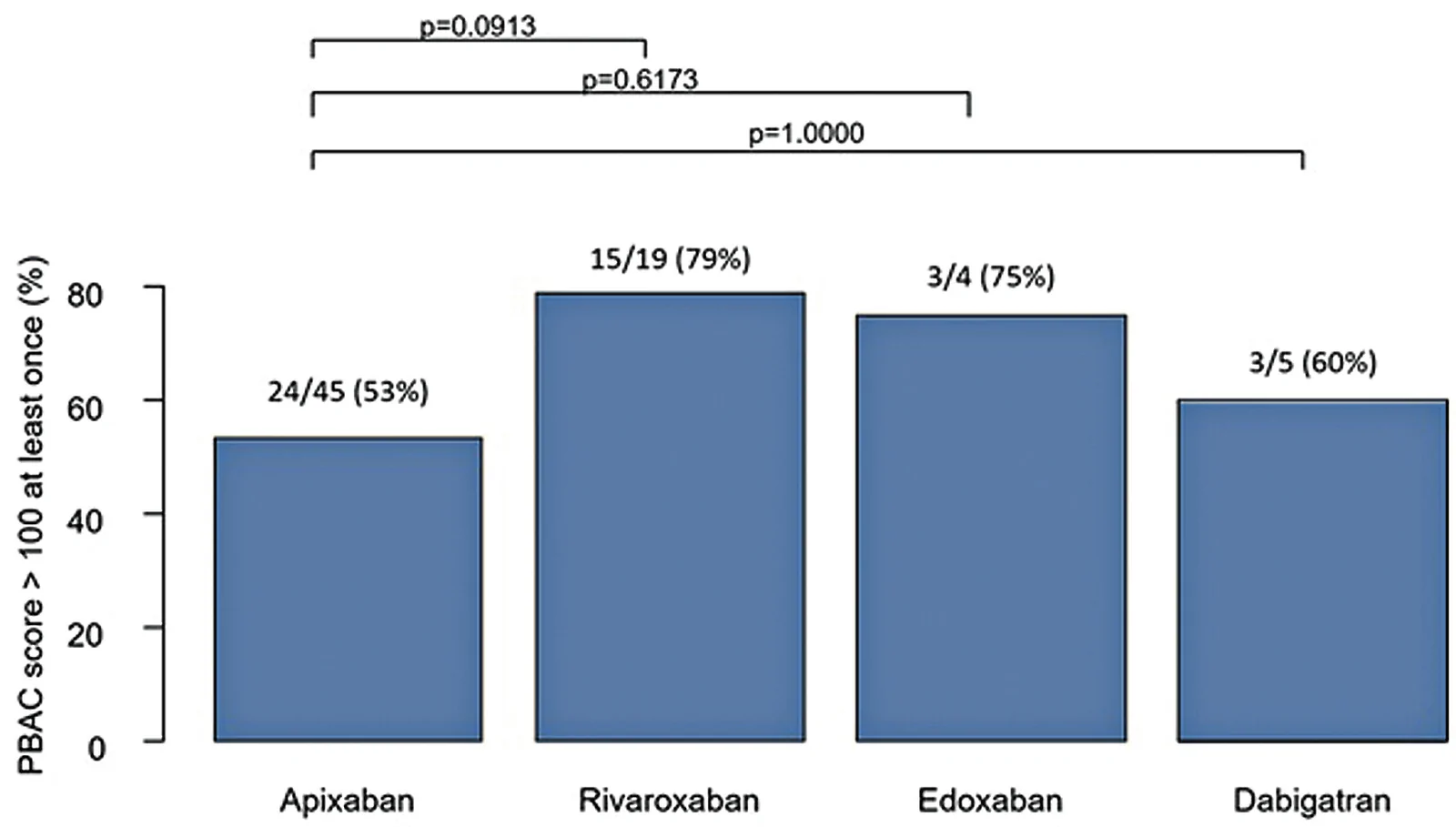
Barplots of the percentage of patients with heavy menstrual bleeding (PBAC score > 100 points) at least once during the observation period. The exact Fisher test was used for comparisons ( *p* -values). PBAC, pictorial blood assessment chart.

PBAC scores were evaluated after 1, 2 and 3 months. Overall, 213 monthly assessments were analyzed in 73 patients. Because of the limited sample size, the primary analysis focused on all single measurements of the PBAC scores using a linear mixed-effects model of log-transformed PBAC scores. Overall, PBAC scores in the rivaroxaban group (mean: 145 points, 95% CI: 103–204 points) were significantly increased by 54% compared with the apixaban group (mean: 93 points, 95% CI: 73–118 points, *p* = 0.0193; Fig. 2 ). There were no significant differences in the edoxaban group and the dabigatran group ( *p* = 0.2192 and *p* = 0.9384, respectively) compared with the apixaban group. These results were also confirmed in a sensitivity analysis excluding the six patients who had changed anticoagulation before study inclusion due to increased menstrual bleeding (the PBAC score in the rivaroxaban group significantly increased by 47% compared with the apixaban group, *p* = 0.0426; no significant results for the edoxaban group and the dabigatran group compared with the apixaban group, respectively). In addition, the incidence of HMB (PBAC scores > 100 points at least once during follow-up) was analyzed as was originally planned. Fifty-three percent of the apixaban group, compared with 79% of the rivaroxaban group, developed HMB, not reaching statistical significance ( *p* = 0.0913; Fig. 3 ).

### Secondary Outcomes with a Potential Influence on HMB

The effects of the intake of NSAIDs on the PBAC scores were analyzed. In a combined model including the treatment groups and differentiating between patients taking NSAIDs or not, the intake of NSAIDs did not have a significant influence on the PBAC scores ( *p* = 0.9548). Similarly, in a univariate model analyzing only the influence of NSAID on the PBAC scores, again, no significant differences were observed ( *p* = 0.9432). Similar results were received for PBAC scores > 100 points ( *p* = 0.9533).

The potential influence of the blood group (all four groups) on HMB was also investigated. Neither in a univariate model nor in a model accounting for the different anticoagulants, an effect of the blood group was observed ( *p* = 0.6663 and *p* = 0.4949, respectively). A comparison of blood group O with the non-O blood groups again showed no significant differences ( *p* = 0.5565 in univariate analysis, *p* = 0.5415 after adjusting for the different anticoagulants).

Even so, the sample size was limited the influence of further parameters on the PBAC scores was tested. Age, ISTH bleeding score at baseline, iron deficiency (defined as ferritin < 13 µg/L), hemoglobin, and the presence of uterine myomas and adenomyosis showed no significant associations with the PBAC scores ( *p* > 0.20 for each evaluation). Hemoglobin levels remained comparable between the baseline visit and visit 1 after 4 months (Wilcoxon signed rank test *p* = 0.174). The laboratory results at baseline were missing in two patients, and after 4 months of follow up laboratory results were not available in two additional patients. With regard to the treatment groups, the mean hemoglobin changed from 13.1 ± 1.2 to 12.8 ± 1.2 g/dL in the apixaban group, from 13.0 ± 0.9 to 13.2 ± 0.9 g/dL in the rivaroxaban group, from 12.5 ± 1.0 to 12.3 ± 0.6 g/dL in the edoxaban group, and from 13.5 ± 0.9 to 13.6 ± 0.3 g/dL in the dabigatran group. There was no significant difference between the treatment groups on hemoglobin at baseline and hemoglobin decrease over time ( *p* > 0.20 at baseline and *p* > 0.20 over time). Repeated measurement correlation between hemoglobin at baseline or hemoglobin decrease with PBAC Scores was −0.10 and −0.06, respectively ( *p* > 0.20 at baseline and *p* > 0.20 over time).

### Duration of Menstrual Bleeding and Frequency of Intracyclic Bleeding

**Fig. 4:**
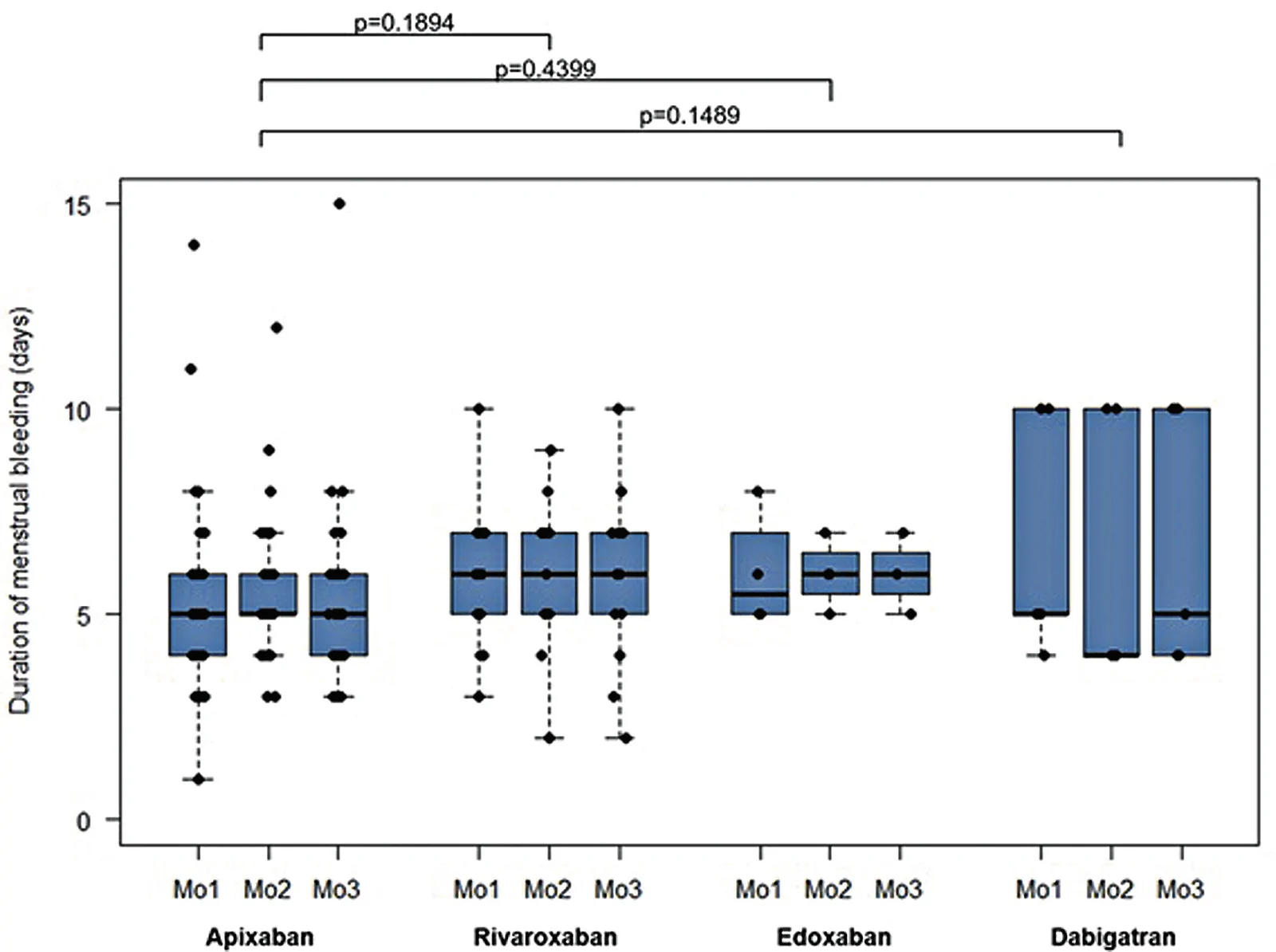
Boxplots of the duration of menstrual bleeding during the observation period (days). Comparisons were performed using a linear mixed effect regression model ( *p* -values) which also provides mean values and 95% CIs: apixaban-group mean: 5.4 days (95% CI: 4.9–5.9), rivaroxaban-group mean: 6.0 days (95% CI: 5.2–6.8), edoxaban-group mean: 6.1 days (95% CI: 4.3–7.9), dabigatran-group mean: 6.6 days (95% CI: 5.0–8.2). Mo, month.

A comparison of the duration of menstrual bleeding in the DOAC treatment groups was performed ( Fig. 4 ). No significant differences between the groups were observed. The mean bleeding duration was shortest during treatment with apixaban (5.4 days *;* 95% CI: 4.9–5.9), followed by rivaroxaban (mean: 6.0 days; 95% CI: 5.2–6.8), edoxaban (mean: 6.1 days; 95% CI: 4.3–7.9) and dabigatran (mean: 6.6 days; 95% CI: 5.0–8.2).

Furthermore, self-reported intracyclic bleedings were assessed, which were more frequently reported in the apixaban group compared with the other DOAC groups (apixaban group 10/45, 22%; rivaroxaban 0/19, 0%; edoxaban 1/5, 20%; dabigatran 0/4, 0%; *p* = 0.0962).

### Results of the (Optional) Vaginal Ultrasound Examinations

**Fig. 5:**
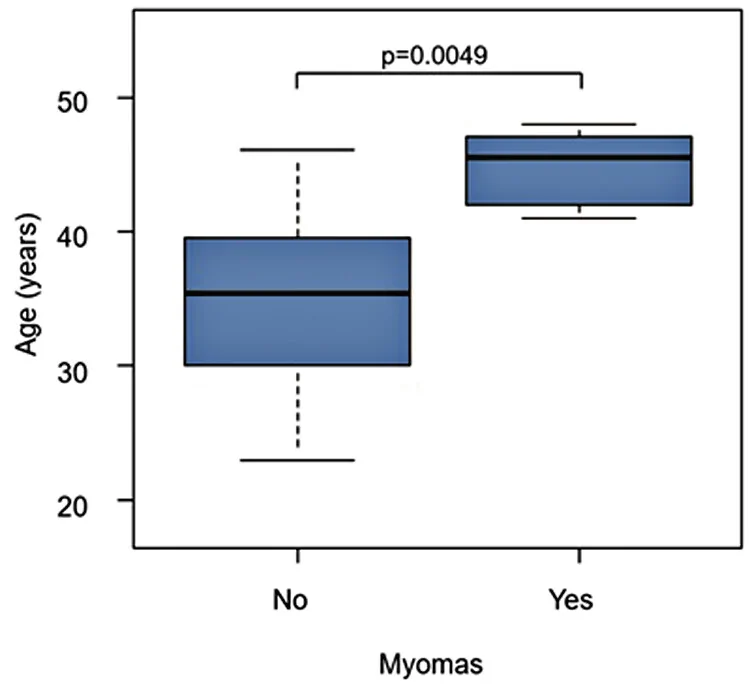
Results of the transvaginal ultrasound in 18 patients. Presence of uterine myomas (6/18, 33%) in dependence on age. The comparison ( *p* -value) was performed using a Mann–Whitney U test.

In 18 of 73 patients (25%), vaginal ultrasound examinations were available. Age was significantly associated with the presence of uterine myomas ( *p* = 0.0049; Fig. 5 ). Overall, 12 myomas in six patients were documented (6/18, 33%). The majority of myomas were localized intramurally without contact to the endometrium ( *n* = 10), while two were localized intramurally with contact to the endometrium.

### Results of the ISTH Bleeding Scores and Additional Bleeding Events during Follow-Up

**Fig. 6:**
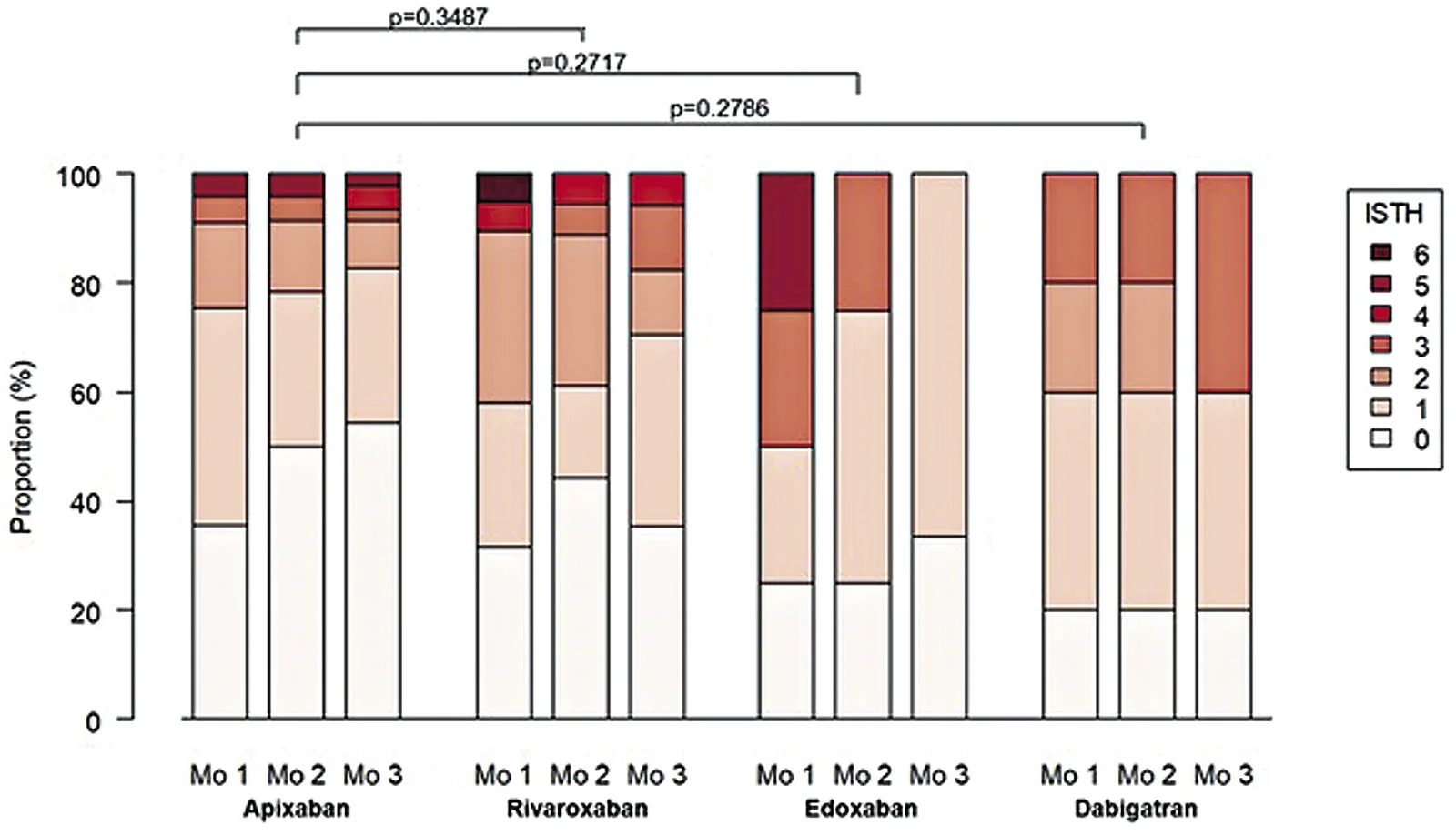
Modified International Society of Thrombosis and Hemostasis (ISTH) bleeding scores during the observation period (normal values in women without anticoagulants < 6 points). Comparisons ( *p* -values) were performed using a mixed-effect ordinal regression model. Mo, month.

The ISTH bleeding scores were documented monthly for 4 months ( Fig. 6 ). No significant differences between the treatment groups were observed.

Additional bleeding events were reported in three patients. In one rivaroxaban -treated patient, HMB and blood deposits on the stool were reported. She received oral iron substitution and spontaneous improvement despite unchanged therapeutic anticoagulation was observed.

In one patient, an asymptomatic minimal intracerebral contusion bleeding occurred after a car accident during treatment with edoxaban. In the third patient, macrohematuria without urinary tract infection was observed during treatment with apixaban. She showed a spontaneous improvement despite continued therapeutic anticoagulation.

## Discussion

Already 10 years ago, de Cem et al were the first to report retrospectively that rivaroxaban treatment increased the duration of menstrual bleeding and patients on rivaroxaban more frequently reported an unscheduled contact with a physician for abnormal uterine bleeding than women using vitamin K antagonists (VKA; 41% vs. 25%, *p* = 0.096). They also reported an increased need for menorrhagia-related medical or surgical intervention in the rivaroxaban group (25% vs. 7.7%, *p* = 0.032). 15 This data was confirmed by Ferreira et al in 2016 in a single-center retrospective study, who published a high incidence of HMB (20%) in women of reproductive age receiving anticoagulation with rivaroxaban. 16

In 2017, Myers et al were the first who compare female patients treated with either rivaroxaban or apixaban. Side effects were monitored prospectively at 1 and 6 months. 5 A total of 139 women aged 55 years or younger were analyzed. Ninety-six were treated with rivaroxaban and 43 with apixaban. Again, HMB was reported in 25% of the women during treatment with rivaroxaban. In contrast, only 9.3% of the women taking apixaban had HMB. Calculated hazard ratio of the likelihood of menorrhagia was 2.688, with 95% confidence limits of 0.989 to 7.273. Although not statistically significant, this result was consistent with higher rates of menorrhagia during treatment with rivaroxaban compared with apixaban. These results are in agreement with the findings of the HEMBLED registry, which is the first cohort study analyzing spontaneous menstrual bleeding during DOAC-treatment not influenced by hormonal contraceptives or IUDs.

The higher rates of HMB during treatment with rivaroxaban may be due to different pharmacokinetics with lower peak and higher trough levels when the anticoagulant is taken twice daily (apixaban) instead of once daily (rivaroxaban). Moldenhauer et al collected data on peak and trough anti-Xa concentrations in patients treated with either rivaroxaban ( *n* = 93) or apixaban ( *n* = 51). While they did not observe differences in apixaban peak levels in female patients compared with male patients, women had significantly higher rivaroxaban peak concentrations compared with men (308.8 ± 178.1 ng/mL vs. 206.4 ± 80 ng/mL, *p* = 0.013). 17

Of note, in post hoc subanalysis of the phase 3 DOAC trials, the incidence of HMB was estimated to be low, but with a likely low sensitivity to detect HMB caused by the lack of a standardized prospective assessment of menstrual blood loss when the trials were performed. 1 2 18 19 20 So far, none of the international bleeding definitions used for the analysis of major bleeding in regulatory studies for anticoagulants includes HMB.

Age, ISTH bleeding score at baseline, iron deficiency, hemoglobin, and the presence of uterine myomas and adenomyosis showed no significant associations with the PBAC scores, which might have been due to the low sample size of included patients. This is in contrast to the results of the BLEED study, which investigated abnormal uterine bleeding in women taking VKAs or DOAC through a retrospective analysis of prospectively collected data. 21 110 women with a median age of 36 years were recruited. PBAC scores significantly correlated with hemoglobin values at baseline and during therapy, with a significant difference in hemoglobin values before and during anticoagulant therapy. In the HEMBLED registry, hemoglobin values before the start of anticoagulation were not available, and the number of included patients was lower than in the BLEED-study. This may explain the different findings.

In the BLEED study, 15.5% of women reported uterine fibroids. Women with self-reported fibroids required more frequent unplanned medical consultations for bleeding during anticoagulation. In our registry, 33% of patients who received transvaginal ultrasound presented with uterine myomas. No significant correlation between HMB and myomas was observed, which may have been due to the lower patient numbers included in the HEMBLED registry.

The HEMBLED registry is subject to all limitations that are typical for observational studies, including patient selection bias and nonstandardized treatment decisions or reporting bias. Moreover, the small number of included patients limits the transferability of the results to the corresponding patient population. Randomized, controlled, and prospective studies with higher numbers of patients are therefore needed to verify our results.

Due to the COVID-19 pandemic, the start of the registry was delayed. Recruitment of patients slowed down and was therefore expected to remain reduced in comparison to the initial study plans. For these reasons, it was decided to extend the recruitment time until December 2023 instead of August 2022 and to reduce the patient number from 250 to 150 patients. Due to very slow recruitment later on and the strict definition of the end of recruitment time, finally, only 73 DOAC-treated patients could be included in the analysis. Therefore, only a power of 35% for the original primary aim was reached when comparing the binary primary endpoint, and it was decided to change the primary objective and to use the PBAC score without dichotomization to obtain a larger power.

## Conclusion

Despite the limitations mentioned above, the lower-than-intended sample size and the fact that we only analyzed menstruating women in Germany without considering the possible ethnic differences in bleeding susceptibility, we provide the first prospective assessment of the risk of spontaneous HMB in DOAC-treated women of childbearing age uninfluenced by hormonal contraceptive use or IUDs. Rivaroxaban-treated patients experienced significantly higher PBAC scores and higher numbers of HMB (PBAC scores > 100 points) compared with apixaban-treated patients.

These findings should be a call to action to increase the awareness of HMB in young female patients treated with DOAC. Physicians treating young female patients with DOAC should be trained in taking a careful gynecological history in those patients before the start of anticoagulation, and routine recording of HMB under DOAC treatment should be included at every outpatient visit.

In addition, uniform international bleeding definitions to better characterize HMB should be established by the international societies and should be prospectively included in future regulatory studies on new oral anticoagulants when major or minor bleeding is assessed. 2

## Notes

**What is known about this topic?**

- HMB is a common complication of therapeutic anticoagulation in premenopausal women with VTE and can occur in up to 70% of women.
- There is increasing data to suggest that the DOAC used for VTE treatment differ in their menstrual bleeding profiles.
- So far, prospective data on the frequency of “spontaneous” HMB in DOAC-treated women of childbearing age unaltered by oral contraceptives or IUDs have not been analyzed.

**What does this paper add?**

- In the prospective HEMBLED registry, spontaneous HMB was more often observed in rivaroxaban-treated patients (79%) compared with apixaban-treated patients (53%).
- Pictorial blood assessment scores were significantly higher in rivaroxaban-treated patients compared with apixaban-treated patients.
