# Supplementary material for: Heavy Menstrual Bleeding in Women Treated with Direct Oral Anticoagulants: Results of the Prospective HEMBLED Registry
Source: Thromb Haemost. 2025 Nov 12;126(9):974–82. doi: 10.1055/a-2724-4458 (PMC13521661; doi:10.1055/a-2724-4458)
Supplement: Supplementary file 1 [file 10-1055-a-2724-4458_29337308.pdf]

Supplementary materials

Additional informations about the modified PBAC-Score used in the registry

PBAC-Scores were translated into German for better understanding.
The translated version of the modified PBAC score has already been used in our department of coagulation disorders in clinical routine for 5 years before the registry was started.
It had been accepted very well by all doctors and patients during that time and before start of our prospective registry.

*The modified version was used because the original version of the PBAC Score published 35 years ago only contained pictures of tampons or pads with varying degrees of absorbency,without taking into account the different absorbency levels of pads and tampons that are nowadays available. Only two types of tampons and pads had been used by the menstruating women in this primary publication (*Tampas and Kotex Fems super plus tampons and Kotex Simplicity size 2 towels; see Higham JM, O'Brien PM, Shaw RW. Assessment of menstrual blood loss using a pictorial chart. Br J Obstet Gynaecol. Aug 1990;97(8):734-9. doi:10.1111/j.1471-0528.1990.tb16249.x).
We have therefore adapted the modified PBAC score to the menstrual products currently available. Different numbers of blood drops instead of symbols for pads or tampons were used to better define the different sizes of tampons and pads used and thus also the amount of menstrual blood loss.
During their first visit participants were instructed by their physicians or study nurses how to use the PBAC scores. During the follow Up visit the PBAC-score sheets were collected and checked together with the individual participant whether they had been filled in and the points had been added up correctly.

An example of the PBAC-sheet used is given here:

Thrombosis and Haemostasis | DOI 10.1055/a-2724-4458 | © The Author(s).

Thieme

Erfassungsbogen Menstruation

Patienten-Nr: ______________________ Geb.-Jahr: ______________

Alter: ________Jahre Größe: _________cm Gewicht: ___________kg

☐ Kontrazeptiva (Pille) ☐ Spirale ☐ Zwischenblutungen

Hygieneprodukte: Typ der Binden oder Tampons

Die Saugfähigkeit Ihrer Hygieneprodukte erkennen Sie am Tröpfchensymbol auf der Packung.

💧 oder 💧💧 Typ 1 ☐
💧💧💧 oder 💧💧💧💧 Typ 2 ☐
💧💧💧💧💧 oder 💧💧💧💧💧💧 Typ 3 ☐

Falls Sie keinen Tröpfchensymbole finden, entnehmen Sie bitte der Produktbezeichnung, ob das Produkt ausgelegt ist für:

- Leichte Blutungen (Typ 1)
- Mittelstarke Blutungen (Typ 2)
- Starke Blutungen (Typ 3)

Füllmenge der Hygieneprodukte: Datum von: ______________ bis: ______________

Tag:

Tampons	1	2	3	4	5	6	7	8	9	10	Faktor	Summe
💧 oder 💧💧											x1	
💧💧💧 oder 💧💧💧💧											x5	
💧💧💧💧💧 oder 💧💧💧💧💧💧											x10	
Gerinnsel/ durchnässt												
											Summe:	

Tag:

Binden	1	2	3	4	5	6	7	8	9	10	Faktor	Summe
💧 oder 💧💧											x1	
💧💧💧 oder 💧💧💧💧											x5	
💧💧💧💧💧 oder 💧💧💧💧💧💧											x10	
Gerinnsel/ durchnässt												
											Summe:	

So geht's:

Dokumentieren Sie bitte täglich den Verbrauch Ihrer Hygieneprodukte anhand der Tabellen. Tragen Sie dazu die tägliche Anzahl Binden oder Tampons passend zu den Füllmengen ein.

Es empfiehlt sich, innerhalb der Tabelle eine Strichliste zu führen, zu der Sie jeweils einen Strich hinzufügen, wenn Sie ein Hygieneprodukt benutzt haben.

Berechnung Score:

1) Multiplizieren Sie die Anzahl der Striche mit dem jeweiligen Faktor.
2) Tampon oder Binde vollständig durchnässt wird jedes Mal zusätzlich mit 5 Punkten gewertet. „Gerinnsel" ab ca. 2,5 cm mit 5 Punkten; kleiner mit 1 Punkt.
3) Addieren Sie beide Summen, um den Score zu erhalten. Bei einem Score über 100 ist eine Abklärung zu empfehlen.

Thrombosis and Haemostasis | DOI 10.1055/a-2724-4458 | © The Author(s).
